# Toxicity of Calcium Hydroxide Nanoparticles on Murine Fibroblast Cell Line 

**Published:** 2014-12-24

**Authors:** Omid Dianat, Sina Azadnia, Mohammad Ali Mozayeni

**Affiliations:** a * Iranian Center for Endodontic Research, Department of Endodontics, Dental School, Shahid Beheshti University of Medical Sciences, Tehran, Iran; *; b * Dental School, Shahid Beheshti University of Medical Sciences, Tehran, Iran*

**Keywords:** Calcium Hydroxide, Cytotoxicity, Murine Fibroblast, Nanoparticles

## Abstract

**Introduction:** One of the major contributing factors, which may cause failure of endodontic treatment, is the presence of residual microorganisms in the root canal system. For years, most dentists have been using calcium hydroxide (CH) as the intracanal medicament between treatment sessions to eliminate remnant microorganisms. Reducing the size of CH particles into nanoparticles enhances the penetration of this medicament into dentinal tubules and increases their antimicrobial efficacy. This *in vitro* study aimed to compare the cytotoxicity of CH nanoparticles and conventional CH on fibroblast cell line using the Mosmann’s Tetrazolium Toxicity (MTT) assay. **Methods and Materials:** This study was conducted on L929 murine fibroblast cell line by cell culture and evaluation of the direct effect of materials on the cultured cells. Materials were evaluated in two groups of 10 samples each at 24, 48 and 72 h. At each time point, 10 samples along with 5 positive and 5 negative controls were evaluated. The samples were transferred into tubes and exposed to fibroblast cells. The viability of cells was then evaluated. The Two-way ANOVA was used for statistical analysis and the level of significance was set at 0.05. **Results: **Cytotoxicity of both materials decreased over time and for conventional CH was lower than that of nanoparticles. However, this difference was not statistically significant (*P*>0.05). **Conclusion: **The cytotoxicity of CH nanoparticles was similar to that of conventional CH.

## Introduction

The main goal of root canal treatment (RCT) is elimination of the microorganisms, cleaning and shaping/filling of the canals to prevent reinfection. Despite great advances in endodontics, we still witness many cases of treatment failure. One main cause of endodontic treatment failure is the residual microorganisms in the root canal system and periradicular tissues [1]. Evidence shows that inadequate preparation of the root canal system and presence of the residual pathogenic microorganisms are the most important causes of failed endodontic treatment [2].

Despite the availability of various root canal preparation techniques including mechanical debridement and chemical irrigation, achieving a bacteria-free environment in an infectious tooth, is challenging if not impossible. In most cases, the residual microorganisms in the dentinal tubules are capable of recolonization and disruption of the process of healing in the periapical tissues [3]. Most of the intracanal bacteria are eliminated from the root canal system by mechanical preparation. However, due to the complexity of the canal spaces, bacteria inside the dentinal tubules are not completely eliminated by the aforementioned conventional techniques [4] and different intracanal medicaments are often used between the treatment sessions to eradicate the necrotic residues and intracanal microorganisms [5]. Several medications have been recommended for this purpose. Calcium hydroxide (CH) has been used for years by the majority of dentists as the intracanal medicament of choice to eliminate pathogenic intracanal microorganisms [6-10]. Intracanal medicaments must have low toxicity and high biocompatibility because they can easily pass through the apex and contact the periapical soft and hard tissues [11]; in case of toxicity, they can cause inflammation and delay tissue healing [12].

CH was first introduced to the dental market in 1920 and is currently used for inducing the formation of dentinal bridge, apexification and as an intracanal medicament [13]. Applications of CH are due to its antimicrobial effects, ability to neutralize microbial byproducts, tissue dissolution, inhibition of root resorption and induction of hard tissue formation, most of which are because of its alkaline pH [6-10]. Due to the aforementioned biological properties, CH has been recommended for use in many cases of endodontic treatment [14]. Its antibacterial activity against microorganisms and their elimination from the root canal system have been demonstrated in many studies [8]. 

The mechanism of the antimicrobial activity of CH is via its quick decomposition into calcium and hydroxyl ions and consequent creation of a high pH environment that inhibits the enzymatic activities required for microbial metabolism, growth and proliferation [15]. These enzymes are located in the cell membrane of microorganisms and their inactivation by ions released from CH chemically changes the organic components and impairs the supply of nutrients; causing eventual toxic effects on microorganisms [16]. In addition, CH can dissolve organic debris and prevent root resorption. It has been demonstrated that hydroxyl ions can enter the dentinal tubules and reach the external root surface within 7 days. Thus, CH has been recommended to remain in the RCS as an intracanal medicament for 7 days [12]. However, due to high pH, CH is potentially toxic and can cause soft tissue destruction in high doses; this issue may lead to chronic inflammation and necrosis in clinical application [17]. On the other hand, CH may not be capable of eliminating microorganisms in the clinical setting; attributed to its incapability to access some infected areas and the buffering capacity of dentine and tissue fluids [18, 19].

CH has been manufactured in different forms. Some manufacturers have made some modifications in its appearance (dry powder, thick paste, paste in syringe, gel, *etc*.). However, dry powder is the most commonly manufactured form of CH, which requires mixing with chlorhexidine, analgesic solution, sterile saline, water or glycerin prior to application.

Microorganisms like *Enterococcus faecalis* (*E. faecalis)* can lodge into the tubules and take refuge from the antibacterial effects of intracanal medicaments. In order to have adequate efficacy, CH must be able to penetrate into the dentinal tubules so that it can be in direct contact with the microorganisms [20]. Komabayashi *et al.* [21], demonstrated that size of most particles of conventional CH range from 1 to 10 μm; while the diameter of dentinal tubules is approximately 2-2.5 μm near the pulp. They concluded that due to large size, conventional CH particles could not well penetrate into the dentinal tubules. Thus, minimizing the particle size and production of CH in the form of nanoparticles may enable better penetration of medicament into the dentinal tubules and subsequently greater efficacy in elimination of microorganisms since the paste can remain in the tubules for longer periods. As a result, CH nanoparticles will probably show greater antibacterial activity [22]. 

Several strategies have been used to eliminate the shortcomings of CH. For instance, nanoparticulate CH was recently synthetized for dental applications as described earlier by Roy and Bhattacharya [22] with some modifications. Since no study has evaluated the toxicity of this material so far, the purpose of the current study was to compare the cytotoxicity of CH nanoparticles and conventional CH on L929 murine fibroblast cell line using the Mosmann’s Tetrazolium Toxicity (MTT) assay. 

## Materials and Methods

This *in vitro* study was approved in the ethics committee of Shahid Beheshti University of Medical Sciences (Grant no.: 1087) and was conducted in the following stages:

***Preparation of specimen***

Conventional CH (Merck, Whitehouse Station, NJ, USA) and CH nanoparticles (Polymer Institute, Tehran, Iran) with dimensions smaller than 100 nm and 99% purity, both in the form of powder, were used in this study. First, the powders were separately mixed with sterile saline solution (1 gr of powder with 1 cc of saline solution) and transferred to the capillary tubes measuring 10 mm in length for later exposure to L929 fibroblast cells. 

***Culture of L929 fibroblast cell-line***

A cryotube of L929 murine fibroblast cell-line was procured from the Pasteur Institute, Tehran, Iran and after defrosting, cells were cultured in cell culture flasks. Cell culture medium (GIBCO, Grand Island, NY, USA) was complemented by adding 100 international unit/mL of penicillin (Sigma Aldrich, St Louis, MO, USA), streptomycin (Sigma Aldrich, St Louis, MO, USA) and 10% fetal bovine serum (FBS) (PAA, Pasching, Austria). After ensuring cell viability by Trypan Blue dye, cells were counted using a Neubauer counting slide. A total of 10000 cells were transferred to each well of a 24-well plate as mono-layer. For each of the two understudy materials, 10 wells of 3 plates (for different time points of 24, 48 and 72 h) were allocated. Five wells were allocated for the positive controls and 5 others for the negative controls.

***Exposure of fibroblasts to experimental materials***

L929 fibroblasts were placed in each well of a 24-well plate and after 24 h of incubation (5% CO_2_, 98% humidity and 37^°^C temperature), the overlaying culture medium was extracted and 10 specimens of conventional CH and 10 specimens of nanoparticulate CH were exposed to cells for the desired time period in the test wells. The 5 positive control wells contained complete culture medium while the 5 negative control wells contained the complete culture medium along with sodium hypochlorite (as a cytotoxic agent).

**Figure 1:**
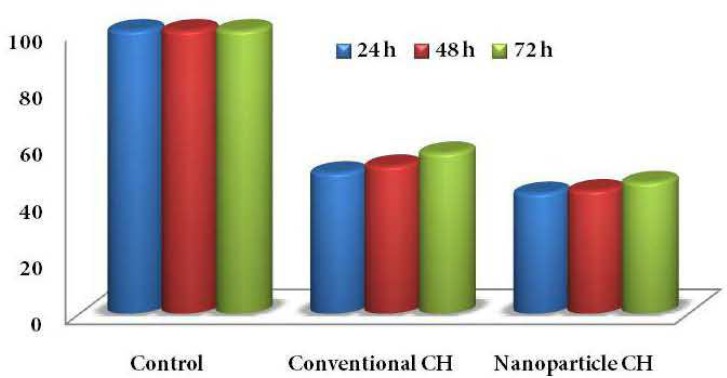
Cell viability at different time points in each study group

***Assessment of the viability of fibroblasts***

The viability of cells exposed to conventional and nanoparticulate CH was assessed at 24, 48 and 72 h using the MTT assay. After the completion of the respective time period, cell culture media were removed from the incubator and one-tenth volume of MTT solution (5 mg/mL, Sigma Chemical Co., St Louis, MO, USA) was added to each well. The plate was then incubated for 4 h (5% CO_2_, 98% humidity and 37^°^C temperature). After completion of the 4-h time period, the plate was removed and the overlaying culture medium was replaced with acidified isopropanol. By doing so, the violet formazan crystals formed in viable cells were dissolved and created a homogenous colored solution. The colored solution was transferred to the ELISA plate and its absorbance was read at 570 nm wavelength with 620 reference filters by ELISA Reader (Anthos 2020, Biochrom, Cambridge, UK). Also, cell viability percentage was calculated by dividing the mean optical density (OD) of the respective group by the mean OD of the positive control group at the same time point multiplied by 100. 

The OD of the well and also the cell viability was statistically analyzed using the two-way ANOVA analysis. Also, the post-hoc Tukey test was used for pairwise comparison. The level of significance was set at 0.05.

## Results

This study was conducted on two experimental groups of 10 each, a positive control group of 5 and a negative control group of 5 samples. A total of 30 samples of the medicaments were in contact with L929 fibroblasts at 24, 48 and 72-h time points. The OD of different groups is summarized in Table 1.

The results showed that the interaction effect of time and group on OD at all three time points was not significant (*P*=0.900). The Tukey’s test revealed no significant differences between the cytotoxicity of conventional or nanoparticulate CH after 24 and 48 h (*P*=0.491). However, the cytotoxicity of both conventional and nanoparticulate CH at 72 h significantly decreased compared to other time points (*P*=0.0001). Control samples showed no significant difference in OD at 24, 48 and 72-h time points after exposure of the culture medium to understudy materials (*P*=0.291).

Figure 1 compares the cell viability of two groups in different time intervals. Accordingly, the lowest percentage of cell viability compared to positive control belonged to CH nanoparticles at 24 h (42.2%) while the highest percentage was seen in conventional CH group at 72 h (56.5%).

## Discussion

This study aimed to compare the biocompatibility of nanoparticulate CH and conventional CH under *in vitro* conditions on L929 murine fibroblasts at 24, 48 and 72 h.

Several factors can influence the success and failure of endodontic treatment. Adequate cleaning and shaping of the canals can eliminate the bacteria to a great extent [23]. In case of efficient canal preparation, the need for intracanal medicaments is minimized. However, achieving this goal is difficult, if not impossible. Therefore, many researchers believe that application of intracanal inter appointment medicaments is necessary [18]. During the recent years, intracanal medicaments have undergone numerous modifications in terms of form and chemical composition. Biologically, cytotoxicity is important for their clinical application. Having significant antibacterial efficacy can lead to efficient destruction of microorganisms as well as inflammation and tissue reactions. Nanoparticulate CH has been recently manufactured to eliminate the shortcomings of conventional CH and enable further penetration of nanoparticles of medicament into the dentinal tubules. 

Among *in vitro* methods, quantitative methods of cell culture have high sensitivity for assessment of the cytotoxicity of materials. Cells used in endodontic research include primary cells for culture such as fibroblasts [24], epithelial cells and lymphoblasts or HeLa cells (Helacyton gartleri; an immortal cell line derived from cervical cancer cells taken from Henrietta Lacks, a patient who eventually died of her cancer) and sarcoma fibroblasts (L939) as monolayer cells [25].

Fibroblasts are the major cells of the connective tissue that are capable of producing and supporting the connective tissue matrix. During the inflammation phase, fibroblasts uptake the inflammatory toxic compounds and bacterial products and eliminate them. Fibroblast cell culture is used for the measurement of cytotoxicity and local reaction of dental materials; these cells are cultured as primary or monolayer cells and are easily procured and cultured with no difference with the primary cells [26]. In our study, fibroblasts were used as monolayer; also, minimum number of cell passage was used in order to preserve the characteristics of cells as much as possible.

**Table 1:** The mean (SD) of optical density (OD) at different time points

	**24 h**	**48 h**	**72 h**
**Conventional CH**	1.06 (0.29)	1.10(0.22)	1.21 (0.19)
**Nanoparticulate CH**	0.90 (0.19)	0.92(0.30)	0.98 (0.10)
**Positive control**	2.13	2.12	2.14
**Negative control**	0.095	0.091	0.093

Cytotoxicity of endodontic materials like other materials in dentistry are first assessed by *in vitro* methods and then, by the *in vivo* tests as the next level. *In vivo* tests have some limitations for the assessment of material properties and mainly assess acute reactions following the application of materials in the oral cavity or investigate their long-term effects. Thus, *in vitro* methods play an important role in assessment of the cytotoxicity of materials; although these methods have their own advantages and disadvantages as well.

In this study, MTT assay was used to evaluate the biocompatibility of intracanal medicaments and their effects on fibroblasts. The MTT assay (3-{4,5-dimethylthiazol-2-yl}-2,5-diphenyl tetrazolium bromide colorimetric assay, *aka *Mosmann’s Tetrazolium Toxicity assay) is a colorimetric assay for assessing cell viability [27]. MTT is a yellow tetrazole, which is absorbed by the mitochondria where it is reduced to purple formazan by succinate dehydrogenase in living cells. An acidified solution is added to dissolve the insoluble purple formazan product into a colored solution [3]. The absorbance of this colored solution can be quantified by measurement of OD at a certain wavelength. By increased reduction of formazan and measurement of OD, cell viability and the cytotoxicity of materials can be measured [28, 29]. 

In a study by Beltes *et al.* [30], the cytotoxicity of three root canal sealers containing CH (Apexit, calcibiotic root canal sealer or CRCS and Sealapex) was compared. All three sealers showed cytotoxicity with Sealapex and Apexit showing the highest and the lowest toxicity, respectively [30]. In their study similar to ours, L929 murine fibroblasts were used and cytotoxicity was assessed at 24, 48 and 72 h. The difference between the two studies was the use of cell counting method instead of the MTT assay and using baby-hamster kidney cells (BHK-21/C13 cells) along with L929 murine fibroblasts. The cytotoxicity of all materials containing CH was confirmed in all cases; this toxicity decreased over time.

Andolfatto *et al.* [31] evaluated the rat subcutaneous tissue reaction to CH-based intracanal medicaments. At 7 days, inflammation in the rat subcutaneous tissue was seen in all three groups; which decreased by day 30. No significant difference was noted in the biocompatibility of different materials. In their study, rat subcutaneous tissue was used and the tested time points were at 7 and 21 days. Moreover, instead of using the MTT assay, morphological and quantitative analyses were carried out following cell staining to measure cytotoxicity. 

Zhan *et al.* [32] compared the biocompatibility of CH and composite resin on fibroblasts derived from human embryonic stem cells, human dental pulp cells and L929 murine fibroblasts and found that the cytotoxicity of both materials decreased over time. In addition, number of viable L929 fibroblasts in CH group at the three time points was less than that in other groups. In their study, L929 murine fibroblasts were also evaluated at 24, 48 and 72 h. The only difference was that instead of the MTT assay, cell counting kit-8 was used for measurement of cell cytotoxicity.

All these studies have attempted to assess the biological effects of different CH compounds or compared their cytotoxicity with that of MTA, composite resin, *etc*.; however, no study has evaluated the effect of structure, shape and size of drug particles in this respect or made a comparison with CH nanoparticles [33].

The biocompatibility of different nanoparticles has been previously assessed and optimal biocompatibility has been reported for some nanoparticles [34]. Undoubtedly, nanoparticles vary in terms of size, shape, concentration, type of surfactant, type of stabilizer, *etc.* [35] and further separate studies are required to assess their biocompatibility.

In a study by Shantiaee *et al.* [25], the cytotoxicity of nanosilver-coated gutta-percha was compared with that of gutta-flow and conventional gutta-percha. The highest toxicity was observed in nanosilver-coated gutta-percha after 1 h. In their study L929 murine fibroblasts and the MTT assay were used, as well. 

Despite the importance of such *in vitro* studies for evaluation of the performance of dental materials, it should be noted that generalization of *in vitro* results to the clinical setting has numerous limitations because in the laboratory environment, materials are applied conveniently and the confounding factors are easily controlled. However, several confounders are present in the oral environment that can be hardly controlled [36, 37]. On the other hand, complex anatomy of the root canal system makes complete debridement almost impossible. Bacteria cannot be completely eliminated even after thorough cleaning and shaping and irrigation using mechanical and chemical techniques [4]. Although complete generalization of *in vitro* results to the clinical setting is not feasible, they are beneficial for the purpose of comparing different drug regimens and screening of materials and techniques. Future *in vitro* and *in vivo* studies are required to assess the biocompatibility of intracanal medicaments in order to cast a final judgment in this regard.

Results of the current study showed that nanoparticulate CH had higher cytotoxicity than conventional CH at all time-points; but this difference was not statistically significant. Such result was obtained by measuring the number of cells that remained viable using the MTT assay and cells were not evaluated morphologically after exposure to these materials. In addition, toxicity of both materials decreased over time. Higher cytotoxicity of nanoparticulate CH may be explained by its better penetration into L929 fibroblasts due to the smaller size of particles [38]. Minimizing the size of particles in most cases increases the efficacy of the material and decreases the required dosage; this is especially important since it also decreases the side effects of the material. Further studies are needed to better elucidate the properties of nanoparticulate CH.

## Conclusion

No significant difference was noted in the cytotoxicity of nanoparticle and conventional CH on L929 murine fibroblast cell-line at all time-points and the cytotoxicity of both materials was found to be similar.
